# Targeting Microglia Using Cx3cr1-Cre Lines: Revisiting the Specificity

**DOI:** 10.1523/ENEURO.0114-19.2019

**Published:** 2019-07-08

**Authors:** Xiao-Feng Zhao, Mahabub Maraj Alam, Yuan Liao, Tingting Huang, Ramkumar Mathur, Xinjun Zhu, Yunfei Huang

**Affiliations:** 1 Department of Neuroscience and Experimental Therapeutics, Albany Medical College, Albany, NY 12208; 2 Department of Molecular and Cellular Physiology; the IBD Center, Division of Gastroenterology, Department of Medicine, Albany Medical College, Albany, NY 12208; 3 Department of Molecular and Cellular Physiology, Albany Medical College, Albany, NY 12208, USA

**Keywords:** cre, cx3cr1, epilepsy, microglia, reporter, tracing

## Abstract

Microglia play a pivotal role in maintaining homeostasis of the CNS. There is growing interest in understanding how microglia influence normal brain function and disease progression. Several microglia-specific Cx3cr1-Cre lines have been developed and have become indispensable tools in many investigations of microglial function. However, some recent studies have reported that these lines may have significant leakage into neurons. Other studies have reported that Cx3cr1 is expressed in non-microglial cells, including neurons and astrocytes, in vitro or in vivo either during brain development or upon neurological insult. All these reports raise serious concerns about the trustworthiness of these Cre-lines and whether the conclusions drawn from previous studies are valid. Here, we found that a floxed fluorescent reporter mouse line which has been frequently used to verify Cre lines displayed spontaneous expression of the GFP reporter, independent of Cre recombinase, thus revealing a potential caveat in assessing cre lines. We further confirmed that two Cx3cr1-Cre mouse lines can drive fluorescent reporter expression largely restrictively in microglia. Finally, we clarified that these two mouse lines maintain microglia-specific expression even following excitatory injury. Together, our findings confirm that two previously created Cx3cr1-Cre lines remain as invaluable tools for studying microglia. Moreover, to ensure the quality of data generated and the soundness of conclusions drawn from such data, it should be compulsory to thoroughly examine reporter lines for spontaneous leakiness when labeling cells to study CNS function and diseases.

## Significance Statement

Microglia-specific Cre-lines are essential for studying the role of microglia in the CNS. Several Cx3cr1-Cre lines have been developed and used in a number of landmark studies. However, there is growing concern in the microglia research community regarding potential leakiness of Cre-lines into neurons. The conclusions drawn from previous studies are also being questioned and key ongoing studies have been stalled. We found that a GFP reporter mouse lines used in a previous study displays spontaneous leakiness into neurons, independent of Cre recombinase. Furthermore, we confirmed that two Cre-lines are microglia-specific and thus can be redeployed without hesitation. Our study also suggests that testing for potential leakiness of GFP reporter lines should be included as a control in cell-tracing experiments.

## Introduction

Microglia are present nearly uniformly throughout the entire central nervous system, where they constantly prune synapses and repair minor injuries. Microglia are also the chief resident immune cells, part of the innate immune response to “danger” signals. Genetic tools that specifically target microglia are essential for unlocking the roles of microglia in the CNS and neurologic diseases.

Endogenous Cx3cr1 is a selective marker for microglia in the CNS, whereas its ligand fractalkine is expressed in neurons. Several mouse lines have been created for studying microglia (Wieghofer and Prinz, 2016). The Cx3cr1-GFP reporter line (stock #005582; The Jackson Laboratory) was created by replacing the coding part of the Cx3cr1 gene with green fluorescent protein (GFP; Jung et al., 2000). This reporter line has been widely used to label microglia for visualization. The expression pattern of Cx3cr1-driven GFP reporter is specific to microglia over the entire period of postnatal brain development and appears to be maintained even following an excitatory injury (Kim et al., 2015). Using a BAC transgenic strategy, two constitutive and two inducible Cx3cr1 promoter-driving cre lines were created (Gong et al., 2003, 2007; Parkhurst et al., 2013; Yona et al., 2013; Peng et al., 2016). The constitutive Cx3cr1-cre line created by Jung et al. (2000; stock #025524; The Jackson Laboratory) was recently reported to have significant leakage into neurons (Haimon et al., 2018; Zhang et al., 2018). Another constitutive Cx3cr1-Cre line was developed by the Mutant Mouse Resource and Research Center (MMRRC). This line was reported to drive microglial-specific expression of a reporter (also created by MMRRC) in the majority of animals examined (Hwang et al., 2017). However, ∼10% of the animals displayed significant leakage into neurons (Hwang et al., 2017). In another study, this line was reported to drive the expression of mT/mG reporter (stock #007676; The Jackson Laboratory) selectively in microglia (Zhao et al., 2018). Two Cx3cr1-CreERT2 inducible lines were created separately by two laboratories. In initial studies, these lines were found to deliver microglial-specific expression (Parkhurst et al., 2013; Yona et al., 2013). However, a recent study found that one of these inducible lines displays significant leakage of the Rosa-Green reporter (stock #007906; The Jackson Laboratory) into neurons (Zhang et al., 2018). It is unclear why the leakage into neurons varies among the different Cx3cr1-cre lines and studies (Parkhurst et al., 2013; Hwang et al., 2017; Zhang et al., 2018; Zhao et al., 2018), but the problem has raised concerns about whether there is a reliable cre-line that targets microglia with good specificity. Moreover, investigators now hesitate to use these lines and some have questioned the validity of the data generated in the previous studies (Parkhurst et al., 2013; Hwang et al., 2017; Zhang et al., 2018; Zhao et al., 2018). Lack of validated cre lines that specifically target microglia would be a significant setback to the field. Therefore, it is imperative to understand how the variable leakiness among these lines is occurring and how it might be rectified. Here, we performed a thorough characterization of three popular GFP reporter lines, along with two Cx3cr1 promoter-driven Cre lines.

## Materials and Methods

### Animals

Male and female mice were housed in a pathogen-free, temperature-controlled, and humidity-controlled facility with a 12/12 h light/dark cycle (lights on at 7 A.M.) and given *ad libitum* access to food and water. All experiments were performed according to the guidelines set by the Institutional Animal Care and Use Committee as well as the National Institutes of Health Guide for the Care and Use of Laboratory Animals. B6.Cg-*Gt(ROSA)26Sor^tm6(CAG-ZsGreen1)Hze^* ^/J^ (hereafter referred to as Rosa-Green^f/f^; stock #007906), B6.Cg-*Gt(ROSA)26Sor^tm14(CAG-tdTomato)Hze^* ^/J^ (hereafter referred to as TdTomato^f/f^; stock #007914), B6.129(Cg)-*Gt(ROSA)26Sor^tm4(ACTB-tdTomato,-EGFP)Luo^* ^/J^(hereafter referred to as mT/mG^f/f^; stock #007676), and B6.129P2(C)-*Cx3cr1^tm2.1(cre/ERT2)Jung^* ^/J^(hereafter referred to as Cx3cr1-CreERT2; stock #020940) were acquired from The Jackson Laboratory. Tg(Cx3cr1-Cre)MW126Gsat/Mmucd (hereafter referred to as Cx3cr1-CreM; stock #036395-UCD) mice were acquired from the MMRRC (Table 1).

### Antibodies, immunohistochemistry (IHC), and acquisition of images

Male and female mice were anesthetized with pentobarbital (100 mg/kg, i.p.) and transcardially perfused with PBS followed by 4% paraformaldehyde (PFA) in PBS, pH 7.4. Brains were post-fixed overnight in 4% PFA buffer, followed by cryoprotection in 30% sucrose in PBS for at least 48 h. Mouse brains were then embedded in Neg-50 frozen section medium (Fisher Scientific) and sectioned using a cryostat at 35 µm for all histologic analyses. Brain coronal sections with similar anatomic locations (near bregma –2 mm position based on the mouse brain atlas) were selected for all histologic analyses. Brain sections were washed with PBS, blocked and permeabilized with 10% BSA (Sigma) and 0.3% Triton X-100 (Sigma) in PBS at room temperature for 1 h. Brains were stained with rabbit anti-GFP (1:2000; catalog #ab290; Abcam), rabbit anti-Iba1 (1:400; catalog #019-19471; Wako), rabbit anti-NeuN (1:400; catalog #24307; Cell Signaling Technology; Subbanna et al., 2018), mouse anti-NeuN (1:250; catalog #MAB377; Millipore; Sahay et al., 2011), and rabbit anti-GFAP (1:500; catalog #AB5541; Millipore) (Table 1). Sections were incubated with primary antibodies overnight at 4°C, and then washed with PBS for 5 min and repeated three times, followed by incubation with appropriate fluorescent-conjugated secondary antibodies for 2 h at room temperature. For brain sections that needed to be stained with goat anti-Iba1 (1:250; catalog #NB100-1028; Novus Biologicals), free-floating sections were permeabilized and blocked with blocking buffer containing 0.3% Triton X-100 and 5% normal donkey serum in PBS at room temperature for 1 h. Sections were then incubated with goat anti-Iba1 diluted in blocking buffer overnight at 4°C, and then washed with PBS for 5 min and repeated three times, followed by incubation with appropriate fluorescent-conjugated secondary antibodies diluted in blocking buffer for 2 h at room temperature. Nuclei were counterstained with DAPI (Sigma) and coverslips were applied with Fluoromount G (Southern Biotech) and sealed with nail polish. Co-immunostaining requires the consideration of the compatibility of antibody host species. Therefore, two pairs of anti-Iba1 and anti-NeuN antibodies have been used to detect microglia and neurons, respectively, in the present study and explicitly noted in the figure legends. Both pairs of antibodies are cell-type specific (Extended Data Fig. 1-3*A*,*B* ). Lycopersicon esculentum lectin was used to label the endothelial cells of the blood vessels (Mazzetti et al., 2004). All representative images were acquired using a Zeiss LSM 880 confocal microscope with Airyscan and processed with Zen black 2.1 or Zen blue lite 2.3 (Carl Zeiss). Images were acquired using the tiles and positions module and a 25× water objective lens. Each tile represents a z-stack of 15 images at 1-µm intervals acquired from a single position, and images (tiles) from a series of positions were stitched together, followed by maximal intensity projection to form a 2-D image as presented. For quantification of Iba1-positive, NeuN-positive, and GFAP-positive cells, a full mosaic of entire brain sections was acquired using a Neurolucida imaging system (MBF Bioscience), under a 10× lens and quantified with Neurolucida software. Microglia within the areas of the M1 motor cortex around Layer IV, the hippocampal radiatum layer adjacent to pyramidal CA1, the stratum lucidum adjacent to CA3, and the dentate gyrus (hereafter referred to as cortex, CA1, CA3, and DG, respectively), were quantified. GFP^+^/Iba^+^, GFP^+^/NeuN^+^, and GFP^+^/GFAP^+^ double-positive cells over the entire brain sections were quantified.

### Tamoxifen treatment

Tamoxifen (catalog #T5648; Sigma‐Aldrich) was dissolved in corn oil (catalog #C8267; Sigma‐Aldrich) at a concentration of 20 mg/ml by shaking overnight at 37°C. Dissolved tamoxifen was stored at 4°C for the duration of injections. Cx3cr1-CreERT2J±;mT/mG^f/-^ mice seven to eight weeks old (either sex) were injected intraperitoneally with tamoxifen daily at 100 mg/kg body weight for five consecutive days.

### Pilocarpine treatment

Status epilepticus (SE) was induced by pilocarpine as described in previous studies (Müller et al., 2009). In brief, to minimize the peripheral cholinergic side effects of pilocarpine, 8- to 10-week-old mice of either sex were first injected intraperitoneally with 1 mg/kg methyl scopolamine (S8502; Sigma-Aldrich) in 0.9% NaCl for 10 min before injection of pilocarpine (P6503; Sigma-Aldrich). For all groups, pilocarpine administration was started at the same time of day, 9 A.M. to 3 P.M. We used a modified ramping-up pilocarpine injection protocol (Müller et al., 2009). In brief, the first dose of 200 mg/kg pilocarpine was given to all the mice by intraperitoneal injection and followed by repeated low-dose treatment at 50 mg/kg every 15 min until the onset of stage four or five seizures. Seizures were classified according to the Racine scale (Racine, 1972), with modification made by Borges et al. (2003): stage 0, normal activity; stage 1, rigid posture or immobility; stage 2, stiffened, extended and often arched tail; stage 3, partial body clonus, including forelimb or hindlimb clonus or head bobbing; stage 4, whole body continuous clonic seizures with rearing; stage 5, severe whole-body continuous clonic seizures with rearing and falling; stage 6, tonic-clonic seizures with loss of posture or jumping. Animals were allowed to develop SE for 4 h. SE was terminated by diazepam treatment (10 mg/kg, i.p.), followed by administration of a single dose of dextrose (1.5 g/kg, i.p.). Animals were placed on a 30°C warm pad for recovery for 1 h.

### Statistical analysis

Data were analyzed using GraphPad Prism 7 software and presented as the mean ± SEM.

## Results

### Spontaneous leakiness of Cre-dependent GFP reporter lines in the CNS

**Figure 1.**
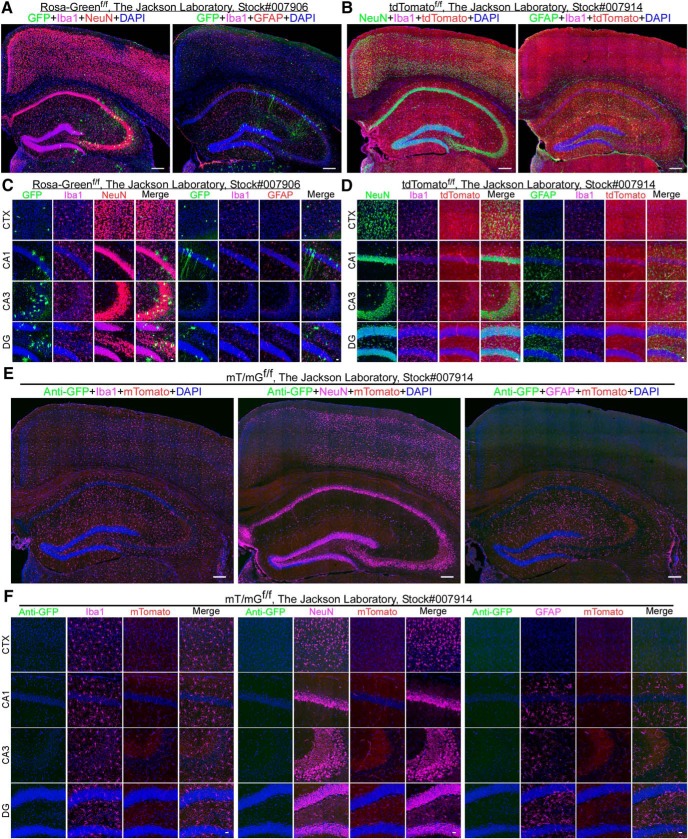
Leakage of fluorescent reporter lines. ***A***, Full montage of confocal images showing GFP expression in the cortex and hippocampus of the Rosa-Green^f/f^ mouse line. Mouse brain sections were stained with: goat anti-Iba1 (pink), rabbit anti-NeuN (red) or chicken anti-GFAP (red), and DAPI (blue). Scale bar, 200 μm. ***B***, Full montage of images showing tdTomato expression in the cortex and hippocampus of the tdTomato^f/f^ mouse line. Mouse brain sections were stained with: rabbit anti-Iba1 (pink), mouse anti-NeuN (green) or chicken anti-GFAP (green), and DAPI (blue). Scale bar, 200 μm. ***C***, High-magnification images showing that the morphology of GFP-positive cells varies among anatomic regions in the cortex (CTX) and the hippocampal CA1, CA3, and DG in Rosa-Green^f/f^ mice. Scale bar, 20 μm. ***D***, High-magnification images showing tdTomato-positive cells in the CTX and the hippocampal CA1, CA3, and DG in tdTomato^f/f^ mice. Scale bar, 20 μm. ***E***, Full montage of confocal images indicating no GFP-positive cells in the CTX and the hippocampus of the mT/mG^f/f^ mouse line. Mouse brain sections were stained with: rabbit anti-GFP (green), goat anti-Iba1 (pink) or mouse anti-NeuN (pink) or chicken anti-GFAP (pink), and DAPI (blue). Scale bar, 200 μm. ***F***, High-magnification images no GFP-positive cells in the CTX and the hippocampal CA1, CA3, and DG in the mT/mG^f/f^ mouse line. Scale bar, 20 μm. Also see Extended Data Fig. 1-1, Fig. 1-2, Fig.1-3.

GFP reporter lines are frequently used to verify the cell specificity of Cre lines. While Cre lines have been blamed for the cause of leakiness into neurons, the reliability of Cre-dependent GFP reporter lines has not been seriously evaluated or even discussed. We started by examining the Rosa-Green^f/f^ reporter line (stock #007906), which has been widely used. This reporter line was recently used to evaluate the microglial specificity of the constitutive B6J.B6N(Cg)-*Cx3cr1^tm1.1(cre)Jung^* ^/J^ (Cx3cr1-CreJ; stock #025524; The Jackson Laboratory) and inducible B6.129P-*Cx3cr1^tm1Litt^* ^/J^ (Cx3cr1-CreERT2, stock#:005582; The Jackson Laboratory) Cre lines (Zhang et al., 2018). In that study, substantial neuronal leakiness of the GFP reporter was reported. We harvested Rosa-Green^f/f^ mouse brains at around two to six months of age and selected three sections from anatomically comparable regions for evaluation. To our surprise, 33.33% of the animals displayed significant expression of GFP reporter throughout the brain, from individually sporadic GFP-positive cells to clusters (Fig. 1*A*,*C*; Extended Data Fig. 1-1*A*). The leakiness varied significantly between the two hemispheres (Extended Data Fig. 1-1*A*) and among animals even in the same age group. Among the GFP-positive cells in the cortex, Iba1^+^, NeuN^+^, and GFAP^+^ populations are 2.80 ± 2.45%, 17.42 ± 15.66%, and 0%, respectively. In the hippocampus, Iba1+, NeuN+, and GFAP+ populations are 0%, 41.71 ± 15.45%, and 0.47 ± 0.83%, respectively. Therefore, a significant portion of GFP-positive cells are Iba1/NeuN/GFAP- triple negative in the cortex (79.8%) and hippocampus (57.8%; two males and one female). Notably, some weak NeuN-positive cells lacked typical neuronal morphology (Fig. 1*C*; Extended Data Fig. 2-1*B*,*C*). the exact identity of these cells needs to be determined.

10.1523/ENEURO.0114-19.2019.f1-1 Extended Data Figure 1-1 Evaluation of the cre-independent fluorescent protein expression of reporter lines. ***A***, ***B***, Full montage images of brain coronal sections showing GFP expression in the Rosa-Green^f/f^ and mT/mG^f/f^ mouse lines. The Rosa-Green^f/f^ mouse brain sections were stained with DAPI. The mT/mG^f/f^ mouse brain sections were stained with rabbit anti-GFP (green) and DAPI. Scale bar, 200 μm. Download Figure 1-1, TIF file.

**Figure 2.**
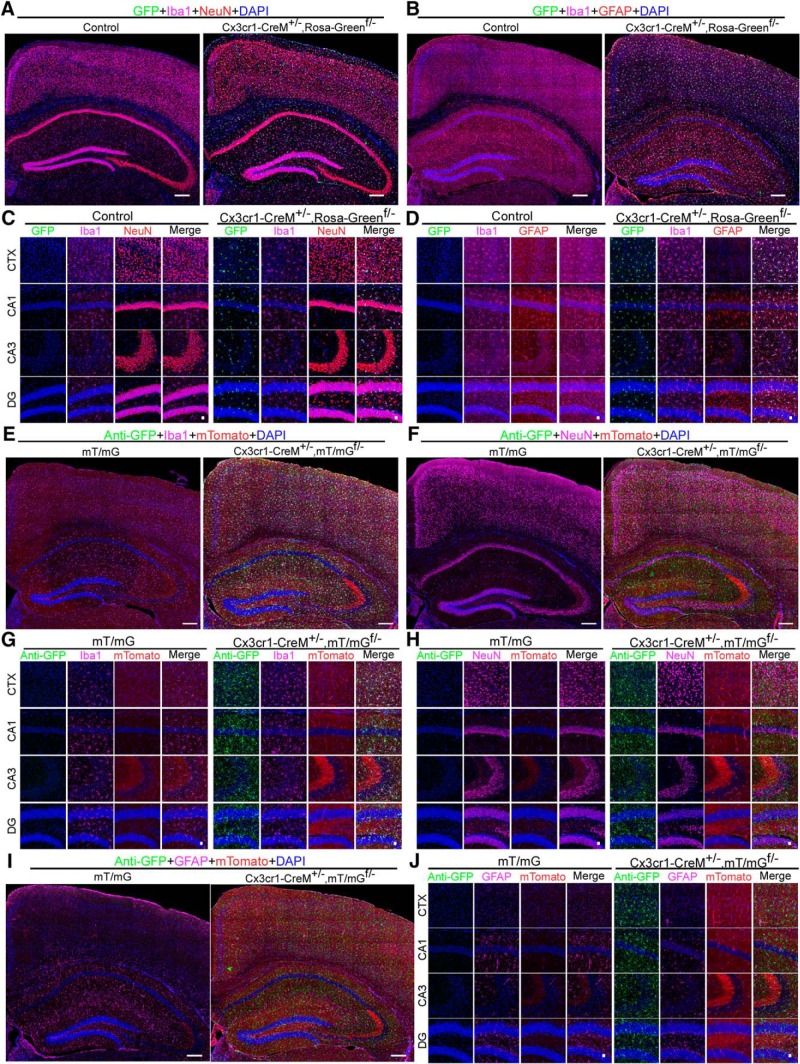
Specificity of Cx3cr1-Cre lines in targeting microglia. ***A***, ***B***, Full montage of confocal images showing GFP expression in the CTX and the hippocampus of Cx3cr1-CreM±;Rosa-Green^f/-^ mice. Brain sections were triple-stained with goat anti-Iba1 (pink), rabbit anti-NeuN (red) or chicken anti-GFAP (red), and DAPI (blue). Scale bar, 200 μm. ***C***, ***D***, High-magnification images from CTX, CA1, CA3, and DG. Scale bar, 20 μm. ***E***, ***F***, ***I***, Full montage of confocal images showing GFP expression in the CTX and the hippocampus of mT/mG^f/-^ and Cx3cr1-Cre±;mT/mG^f/-^ mouse brains. Mouse brain sections were stained with rabbit anti-GFP (green), goat anti-Iba1 (pink) or mouse anti-NeuN (pink) or chicken anti-GFAP (Pink), and DAPI (blue). Scale bar, 200 μm. ***G–J***, High-magnification images from CTX, CA1, CA3, and DG. Scale bar, 20 μm. Also see Extended Data Fig. 1-3, Fig. 2-1.

10.1523/ENEURO.0114-19.2019.f1-2 Extended Data Figure 1-2 Fluorescent protein expression in blood vessels in mT/mG^f/f^ and tdTomato^f/f^ lines. ***A***, Full montage of confocal images showing mTomato expression in the CTX and the hippocampus of mT/mG^f/f^ mice, respectively. Brain sections were stained with lectin (green) and DAPI (blue). Scale bar, 200 μm. ***B***, High-magnification images from CTX, CA1, CA3, and DG. Scale bar, 20 μm. ***C***, Full montage of confocal images showing tdTomato expression in the CTX and the hippocampus of tdTomato^f/f^ mice, respectively. Brain sections were stained with lectin (green) and DAPI (blue). Scale bar, 200 μm. ***D***, High-magnification images from CTX, CA1, CA3, and DG. Scale bar, 20 μm. Download Figure 1-2, TIF file.

**Figure 3.**
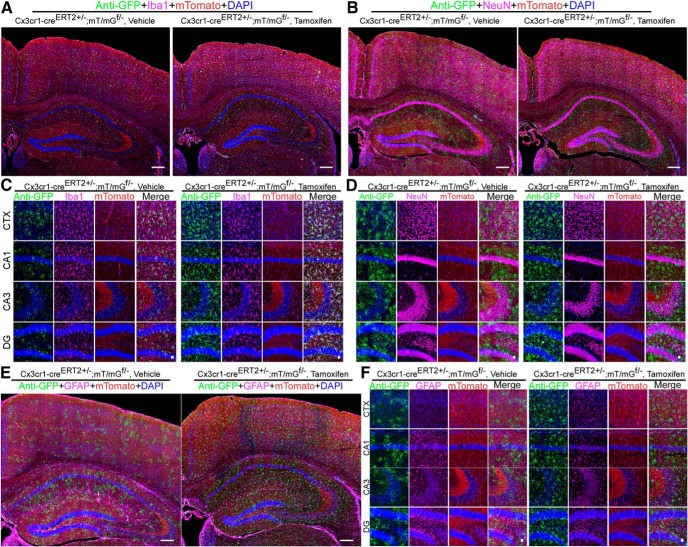
Basal expression of cre recombinase activity and microglial specificity of Cx3cr1-CreERT2 inducible line. ***A***, ***B***, ***E***, Full montage of confocal images showing GFP reporter expression in the CTX and hippocampus of Cx3cr1-CreERT2±;mTmG^f/-^ mice treated either vehicle or tamoxifen. Mouse brain sections were stained with rabbit anti-GFP (green), goat anti-Iba1 (pink) or mouse anti-NeuN (pink) or chicken anti-GFAP (Pink), and DAPI (blue). Scale bar, 200 μm. ***C***, ***D***, ***F***, High-magnification images from CTX, CA1, CA3, and DG. Scale bar, 20 μm. Also see Extended Data Fig. 1-3.

10.1523/ENEURO.0114-19.2019.f1-3 Extended Data Figure 1-3 Specificity of anti-Iba1 and NeuN antibodies used in this study. ***A***, Confocal images showing both goat anti-Iba1 (green) and rabbit anti-Iba1 (red) antibodies can selectively label microglia in control mice. ***B***, Confocal images showing both mouse anti-NeuN (red) and rabbit anti-NeuN (green) antibodies can selectively label neurons in control mice. Download Figure 1-3, TIF file.

10.1523/ENEURO.0114-19.2019.f2-1 Extended Data Figure 2-1 Specificity of the Cx3cr1-creM^+/-^ mouse line. ***A***, Full montage of brain coronal sections showing GFP reporter expression in microglia in Cx3cr1-creM^+/-^;Rosa-Green^f/-^ mice. Mouse brain sections were stained with goat anti-Iba1 (red) and DAPI (blue). Scale bar, 200 μm. ***B***, Brain sections of Cx3cr1-CreM^+/-^;Rosa-Green^f/-^ mice were stained with: goat anti-Iba1 (pink), rabbit anti-NeuN (red), and DAPI (blue). Confocal images showing bright, large GFP-positive cells (numbered) that were sporadically present in the cortex and hippocampus. Scale bar, 200 μm. ***C***, High-magnification images of GFP^+^ cells showing that only one out of twelve GFP^+^ cells co-localized with NeuN+ (red) cells. Scale bar, 20 μm. Download Figure 2-1, TIF file.

The tdTomato line is also frequently used for cell lineage tracing. According to The Jackson Laboratory website, this line may express very low levels of red fluorescent protein (tdTomato) in the absence of cre recombinase. In addition, a very small portion of the animals was found to be very leaky, leading to the whole body becoming pinkish. Accordingly, we evaluated this reporter line. We found that the seven animals examined display significant leakage of tdTomato in the brain (four males and three females; Fig. 1*B*,*D*). Among the tdTomato positive cells in the cortex, Iba1^+^, NeuN^+^, and GFAP^+^ populations are 2.00 ± 4.5%, 4.00 ± 8.9%, and 0%, respectively. In the hippocampus, Iba1+, NeuN+, and GFAP+ populations are 0, 44 ± 0.9%, 0.70 ± 1.6%, and 0.88 ± 1.2%, respectively. It appears that tdTomato predominantly labels vascular structures in the CNS. Accordingly, we employed lycopersicon esculentum lectin (Mazzetti et al., 2004) to label endothelial cells in the blood vessels (Extended Data Fig. 1-2*A*,*B*). We observed that tdTomato signal is co-localized with lectin, particularly in the hippocampus. This data suggests that there is a significant leakage of tdTomato expression into the blood vessels in the CNS in the absence of cre recombinase.

The mT/mG reporter line is most frequently used in cell lineage tracing. Mice are expected to express membrane tagged-Tomato (mTomato) ubiquitously independent of cre recombinase whereas the expression of membrane tagged-GFP(mGFP) requires cre-mediated homologous recombination. Indeed, mTomato is expressed in various brain structures but is noticeably present in the hippocampal CA3 area and in the blood vessels (Extended Data Fig. 1-2*C*,*D*). We saw that lectin is colocalized with mTomato, confirming that mTomato is highly expressed in the blood vessels. We employed an anti-GFP antibody to detect the expression of mGFP. We found no leakage of mGFP expression over the entirety of the sections from all seven mT/mG^f/f^ mice we examined (three males and four females; Fig. 1*E*,*F*; Extended Data Fig. 1-1*B*). Therefore, we conclude that the mT/mG^f/f^ line is a reliable reporter for evaluating Cre lines in the CNS.

### The Cx3cr1-creM mouse line mediates microglial expression of floxed GFP reporters

Heterozygous Cx3cr1-CreM± mice were acquired from the MMRRC. This line has been maintained in a heterozygous state by crossing with wild-type mice. We mated Cx3cr1-CreM± mice with mice homozygous for the Rosa-Green^f/f^ reporter to generate Cx3cr1-CreM±;Rosa-Green^f/-^ mice. Brains were harvested from the latter at around two months of age. We found that 99.2 ± 0.3% and 99.8 ± 0.16% of microglia are GFP-positive in the cortex and hippocampus, respectively (two males and three females; Fig. 2*A–D*). However, we also observed several dozens of non-microglial GFP-positive cells scattered sporadically throughout the entire brain (Fig. 2*A–D*; Extended Data Fig. 2-1*A–C*). NeuN^+^/GFP^+^ and GFAP^+^/GFP^+^ populations are 0.13 + 0.5% and 0%, in the cortex, and 0% and 0.2 ± 0.5% in the hippocampus, respectively. These non-microglial GFP-positive cells likely result from leakage of the Rosa-Green reporter itself. The specificity of the Cx3cr1-CreM line was further verified in Cx3cr1-Cre±;mT/mG^f/-^ mice (Fig. 2*E–J*). We observed that among the GFP-positive cells, 99.9 ± 0.17% and 99.7 ± 0.36% were Iba1 positive in the cortex and hippocampus, respectively (Fig. 2*E*,*G*); none of GFP^+^ cells are either NeuN-positive or GFAP-positive (two males and two females; Fig. 2*F*,*H–J*). Taken together, these data suggest that the Cx3cr1-CreM± line drives microglial-specific expression of floxed GFP reporters.

### Leakiness of the inducible Cx3cr1-creERT2 mouse line

A recent study reported that the Cx3cr1-CreERT2 line (Parkhurst et al., 2013) has a significant leakage into neurons (Zhang et al., 2018). Another Cx3cr1-CreERT2 mouse line was developed by Jung et al. (2000). This prompted us to determine whether this line, too, has any leakiness into neurons. We crossed this inducible line with an mT/mG^f/f^ reporter line and mGFP expression was detected by anti-GFP antibody. We found that Cx3cr1-CreERT2J±;mT/mG^f/-^ mice displayed significant expression of GFP reporter in microglia even without tamoxifen treatment (one male and two females; Fig. 3*A–F*). In vehicle treated mice, up to 18.9 ± 4.3% and 23.0 ± 3.4% of Iba1^+^ cells are GFP positive in the cortex and hippocampus, respectively. After tomaxifen treatment, all Iba1^+^ cells are GFP positive (two males and one female). Again, none of GFP positive cells are either NeuN- or GFAP- positive. These data suggest that in this Cx3cr1-CreERT2J mouse line, Cre-ERT2 recombinase possesses some basal enzymatic activity independent of tamoxifen. However, the Cre-dependent expression is nearly completely restricted to microglia (Fig. 3*A–F*).

### Transient Cx3cr1 activity in neurons and astrocytes following excitatory injury

**Figure 4.**
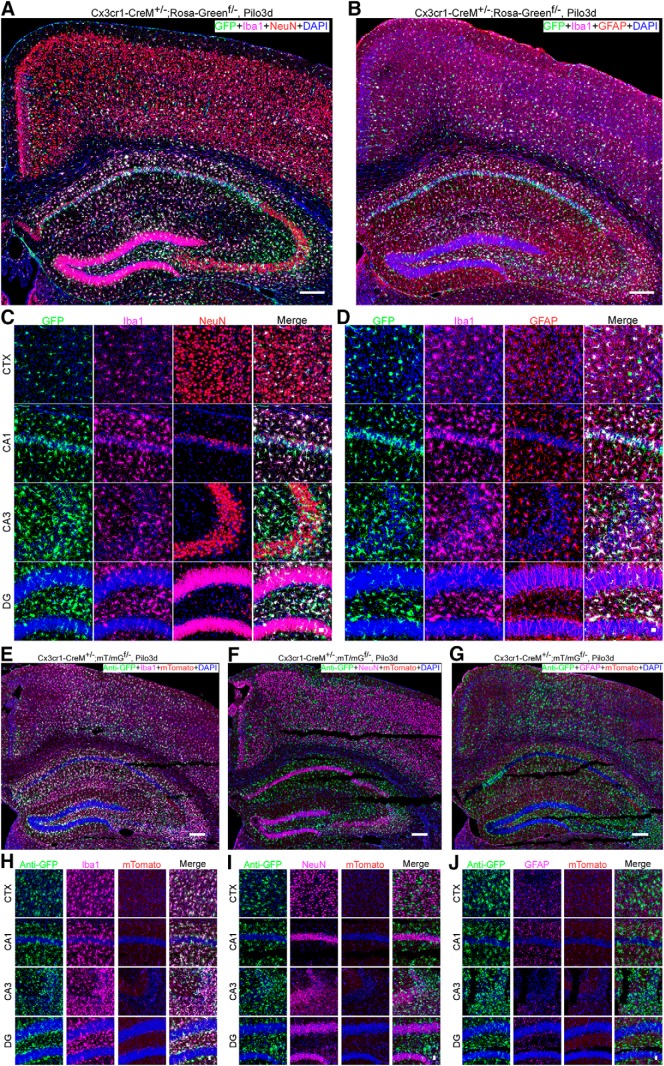
Specificity of Cx3cr1-Cre lines following SE. ***A***, ***B***, Full montage of confocal images showing GFP reporter expression in the CTX and hippocampus of Cx3cr1-Cre±;Rosa-Green^f/-^ mice 3 d post-SE induced by pilocarpine. Mouse brain sections were stained with goat anti-Iba1 (pink), rabbit anti-NeuN (red) or chicken anti-GFAP (red), and DAPI (blue). Scale bar, 200 μm. ***C***, ***D***, High-magnification images from CTX, CA1, CA3, and DG. Scale bar, 20 μm. ***E–G***, Full montage of confocal images showing GFP reporter expression in the CTX and hippocampus of Cx3cr1-Cre±;mT/mG^f/-^ mice 3 d post-SE induced by pilocarpine. Mouse brain sections were stained with rabbit anti-GFP, goat anti-Iba1 (pink) or mouse anti-NeuN or chicken anti-GFAP, and DAPI (blue). Scale bar, 200 μm. ***H–J***, High-magnification images from CTX, CA1, CA3, and DG. Scale bar, 20 μm. Also see Extended Data Fig. 1-3.

Cx3cr1 was reported to be expressed in cultured neurons and induced in neurons and astrocytes following brain ischemia and severe seizures, respectively (Meucci et al., 2000; Yeo et al., 2011; Dworzak et al., 2015; Wang et al., 2018). It was postulated that leakiness into neurons was due to transient expression of Cx3cr1. However, there is no reliable anti-Cx3cr1 antibody available. It is unclear whether the transient induction of Cx3cr1 reported in neurons and astrocytes is real. Moreover, the Cx3cr1-GFP mouse line did not show any leakage of the GFP reporter into neurons and astrocytes under excitatory conditions (Kim et al., 2015). Nevertheless, we evaluated transient Cx3cr1 activity in Cx3cr1-CreM±;Rosa-Green^f/-^ (Fig. 4*A–D*) and Cx3cr1-CreM±;mT/mG^f/-^mice (Fig. 4*E–H*). Animals around eight weeks of age were treated with pilocarpine to induce SE for 4 h. Mouse brains were harvested 3 d post-SE. In Cx3cr1-CreM±;Rosa-Green^f/-^, we found that 98.8 ± 1.1% and 99.6 ± 0.24% of GFP^+^ cells were Iba1-positive in the cortex and hippocampus, respectively (two males and one female). Among the other GFP^+^ cells, only 0.31 ± 0.54% and 0.07 ± 0.13% are NeuN positive in the cortex and hippocampus, respectively and none of them are GFAP-positive (Fig. 4*A–D*). Those non-microglial GFP-positive cells are presumably from the spontaneous leakiness of the Rosa-Green^f/f^ reporter. Moreover, in Cx3cr1-Cre±;mT/mG^f/-^ mice, we observed that nearly all GFP-positive cells were microglia (99.9 ± 0.13% in the cortex and 100% in the hippocampus; two males and one female; Fig. 4*E–H*). We saw few, if any, NeuN^+^/GFP^+^ or GFAP^+^/GFP^+^ double-positive cells. Thus, our data confirm there is little or no transient Cx3cr1-driven Cre activity in neurons or astrocytes in brains that experienced SE.

### Discussion

A reliable microglial-specific Cre line is essential for elucidating the role of microglia in normal CNS function and neurologic disorders. Over the past four to five years, multiple Cx3cr1-Cre lines have been developed for targeting microglia. Unfortunately, leakiness of cre activity into neurons has become a major concern (Zhang et al., 2018). Leakiness is a confounding factor that significantly limits the usefulness and interpretation of the data generated, and greatly discourages the use of these lines. In the present study, we unexpectedly found that the Rosa-Green^f/f^ reporter line used in a previous study by other researchers (Zhang et al., 2018) shows spontaneous leakiness into non-microglial cells in the absence of cre recombinase. This cre-independent leakage likely accounts, at least in part, for the GFP-positive non-microglial cells observed in that previous study (Zhang et al., 2018). We have also confirmed that the Cx3cr1-CreM line from MMRRC is reasonably microglia specific. The inducible Cx3cr1-CreERT2 line developed by Jung et al. (2000), displayed some basal cre activity without tamoxifen treatment, but this expression was restricted to microglia. Finally, we did not find any significant transient Cx3cr1 activity in neurons and astrocytes under the conditions that animals have experienced excitatory injury.

Many cre-dependent fluorescence-based reporter lines have been created for cell-lineage tracing. These reporter lines are also frequently used to evaluate the specificity and recombination efficiency of Cre lines. One would naturally expect that the floxed-fluorescecnt reporter lines are cre dependent. Surprisingly, however, the Rosa-Green^f/f^ and tdTomato^f/f^ lines displayed spontaneous leakiness independent of cre recombinase. Our study suggests that the Rosa-Green^f/f^ reporter line itself could contribute to the leakage into neurons and other cells that was noted by others (Zhang et al., 2018).

In the tdTomato mouse line, we observed cre recombinase-independent leakage predominantly in the hippocampus, which is consistent with what is described at The Jackson Laboratory website, that the donating investigator reported a low level of fluorescent activity in this line. Of note, occasionally, a small fraction of tdTomato mice displayed very broad spontaneous leakage. The entire mouse body can turn pinkish in color. Therefore, caution is needed when implementing this reporter line.

Among the three fluorescent reporters tested, the mT/mG reporter line appears to consistently deliver GFP reporter expression in a cre-dependent manner, not only in the present study, but also in many other studies (Zhao et al., 2018). The exact mechanism of spontaneous leakiness of the fluorescent reporter lines seen in the Rosa-Green^f/f^ and tdTomato^f/f^ lines remains unclear. However, given the variable leakiness of fluorescent reporter lines, it is advisable to include the reporter line(s) as a control, with a thorough analysis.

According to the description on The Jackson Laboratory website, the constitutive Cx3cr1-Cre line created by Jung et al. (2000), displays leakiness into neurons when crossed with a yellow fluorescent protein (YFP) reporter line. No data were provided. The leakiness of this Cx3cr1-Cre line was recently reported, but in conjunction with a Rosa-Green^f/f^ reporter line (Zhang et al., 2018). Given that the Rosa- Green^f/f^ reporter line itself has spontaneous leakiness, this complicates the data interpretation. The YFP reporter line was used to verify two inducible Cx3cr1-CreERT2 lines (Jung et al., 2000; Parkhurst et al., 2013). No leakiness was reported in those studies. If the YFP reporter line has no spontaneous leakiness, then the constitutive Cx3cr1-Cre line developed by Jung et al. (2000), and which is now available at The Jackson Laboratory, is not microglia specific.

In the present study, we verified that the constitutive Cx3cr1-CreM line from the MMRRC is microglia specific. We reached this conclusion based on our data from two reporter lines. Notably, this Cre-line was also deployed in a recent study by Hwang et al. (2017), who found it to drive microglial-specific expression of a reporter. However, the authors also briefly mentioned that ∼ 10% of their animals displayed significant leakage into neurons, but the data were not provided (Hwang et al., 2017). Moreover, in the same study, the GFP reporter line was created by the researchers themselves. Therefore, a thorough study of spontaneous leakiness of Hwang’s reporter line is called for. It will be interesting to see whether their own GFP reporter line has any occasional spontaneous leakage.

The inducible Cx3cr1-CreERT2 mouse line that was developed by Parkhurst et al., displayed a reasonable level of microglial-specificity in their hands (Parkhurst et al., 2013). However, Zhang et al. (2018) reported that this line has significant leakage into neurons. Because the Rosa-Green^f/f^ reporter used by Zhang et al. (2018) displays spontaneous leakage into neurons (as we showed here), further study is required to reconcile the discrepancy. Cx3cr1-CreERT2 developed by Jung et al. (2000) was reported to drive microglia-specific expression. We found that this inducible line can drive expression of GFP reporter in a fraction of microglia before tamoxifen treatment. Thus, the cre activity in this line is not fully dependent on tamoxifen. Nevertheless, tamoxifen treatment led to broad expression of GFP reporter in microglia, without significant leakage into neurons. In the study by Zhao et al. (2018), this line was employed to deplete TSC1 in microglia. On tamoxifen treatment, a fraction of mice displayed spontaneous seizures. In contrast, no spontaneous seizures were observed in the other study (Zhang et al., 2018) that used the Cx3cr1-CreERT2 mouse line developed by Parkhurst et al. (2013). It is unclear whether this Cx3cr1-CreERT2 inducible line has cre activity leakage in the absence of tamoxifen. The partial tamoxifen-independent cre activity observed in the Cx3cr1-CreERT2 line developed by Jung et al. (2000), indicates potential embryonic deletion of TSC1 in a fraction of microglia, which may explain the differences in seizure susceptibility observed in these two studies (Jung et al., 2000; Zhang et al., 2018; Zhao et al., 2018).

All four microglia-targeting Cre lines were created using a BAC strategy, which involves insertion of a large piece of DNA containing not only the gene promoter of interest, but also many other regulatory elements that help to yield the expected expression pattern of the endogenous gene (Gong et al., 2003, 2007). A drawback of this approach is it may cause duplication of the genes brought in within the BAC constructs. This may create issues related to increased gene dosage, which could have unexpected effects, including genetic instability and ectopic gene expression. It is preferable to maintain BAC transgenic lines in a heterozygous state. This should be adopted as a standard practice when handling cre and reporter lines created with BACs.

While the Cx3cr1 gene is described as being very specific to microglia in an unperturbed CNS, several studies have suggested otherwise. Meucci et al. (2000) reported that Cx3cr1is expressed in cultured neurons and promotes neuronal survival. It is unclear how relevant this is to the *in vivo* condition. Wang et al., reported that transient expression of Cx3cr1 in neurons is neuronal protective in an ischemic model (Wang et al., 2018). However, oddly, the IHC data showed that the majority of Cx3cr1-positive cells were neurons and very few, if any, were microglia. Yeo et al. (2011) reported that pilocarpine-triggered SE induced very moderate expression of Cx3cr1 in astrocytes, but again the IHC data are puzzling. Nearly 100% of the Cx3cr1-positive cells were astrocytes. Then, where were the microglia? In the absence of a reliable anti-Cx3cr1 antibody, it is hard to draw any conclusions from these studies. In contrast, our data with reporter lines suggest there is very minimal, if any, transient Cx3cr1 activity in neurons or astrocytes following excitatory injury.

**Table 1.** Materials and reagents

Reagent or resource	Source	Catalog number	RRID
Antibodies			
Rabbit anti Iba1	Wako	019-19471	AB_839504
Goat polyclonal anti Iba1	Novus Biologicals	NB100-1028	AB_521594
Mouse anti-NeuN	Millipore	MAB377	AB_2298772
Rabbit anti-NeuN	Cell Signaling	24307	AB_2651140
Chicken anti-glial fibrillary acidic protein	Millipore	AB5541	AB_177521
Rabbit anti-GFP	Abcam	ab290	AB_303395
chemicals			
Pilocarpine hydrochloride	Sigma	P6503	N/A
(−)-Scopolamine methyl bromide	Sigma	S8502	N/A
Lectin-DyLight 488	Vector Laboratories	DL-1174	AB_2336404
DAPI	Sigma	D9542	N/A
Experimental models: organisms/strains			
ROSA-Green mice	The Jackson Laboratory	007906	IMSR_JAX:007906
mT/mG mice	The Jackson Laboratory	007676	IMSR_JAX:007676
tdTomato mice	The Jackson Laboratory	007914	IMSR_JAX:007914
Cx3cr1-CreERT2 mice	The Jackson Laboratory	020940	IMSR_JAX:020940
Cx3cr1-Cre mice	MMRRC	036395-UCD	MMRRC_036395-UCD
Software and algorithms			
Fiji-ImageJ	Fiji	https://fiji.sc/ or https://imagej.nih.gov/ij/	SCR_002285
GraphPad Prism 7.0	GraphPad Software	https://www.graphpad.com	SCR_002798
Zen Black	Zeiss	https://www.zeiss.com	N/A
Zen Blue	Zeiss	https://www.zeiss.com	SCR_013672
Neurolucida	MBF Bioscience	http://www.mbfbioscience.com	SCR_001775

The source, category, and PRID number of antibodies, chemicals, and mouse lines were provided.

In summary, Cre lines are genetic tools essential for studying gene function in a cell-specific manner. A large array of fluorescent reporter lines has been created for cell lineage tracing and evaluating the cell specificity of many Cre lines. However, the reliability of reporter lines should not be presumed. Our study strongly suggests that both cre and reporter lines need to be thoroughly evaluated.

## Synthesis

Reviewing Editor: Tallie Z. Baram, University of California-Irvine Decisions are customarily a result of the Reviewing Editor and the peer reviewers coming together and discussing their recommendations until a consensus is reached. When revisions are invited, a fact-based synthesis statement explaining their decision and outlining what is needed to prepare a revision will be listed below. The following reviewer(s) agreed to reveal their identity: Jessica Bolton, Sookyong Koh.

Synthesis of reviewers comments and specific issues that require addressing:

Both reviewers found much merit in your work, and believe that it is an important addition to the field, as you propose. However, they felt that prior to publication the paper must be improved as follows:

1. Materials and Methods: The “gold standard” antibody for Iba-1 in the study of microglia is the Wako rabbit antibody. Why did you need to stain some brains with the goat anti-Iba1 antibody? It appears that your GFP antibody was made in rabbit, so perhaps anytime you stained for both GFP and Iba-1, you used the goat Iba-1 antibody? Explanation of this deviation, and notation of which figures show these results, is important for this type of manuscript, which is aiming to validate common tools in the field. Have you validated that the goat and rabbit Iba-1 antibodies show the same pattern of labeling? This would be important to include in the Extended Data, and would be of interest for many readers in the microglia field. The same idea applies for the NeuN antibody-why were 2 different antibodies used, and do they show the same pattern of labeling?

2. Materials and Methods: The statistical analysis section reports that t-tests are used, but I do not see any t-statistics reported anywhere in the results or figure legends. These data seem mostly descriptive (i.e., there are not really experimental conditions imposed, with the exception of the pilocarpine experiments), so it would be better to use chi-square tests to determine whether the % cells labeled as each category differ from what is expected by chance.

3. Results: In the mT/mG line, what do the Tomato vs. the GFP mark? I am unfamiliar with this reporter line, and presume that other readers may be as well, so more description is necessary in the methods and/or results. It appears that the Tomato marks blood vessels, whereas the GFP is Cre-dependent, but it would be helpful to have a description of the distinction between the two colors. If the Tomato labeling of blood vessels is unexpected, then the authors should include an IHC of an endothelial cell marker, such as CD34, to show that this is indeed where the labeling is occurring.

4. Results: The tdTomato reporter line that is included in the Extended Data is very commonly used and far more familiar to me, and thus I believe it should be included in the main Figures. It appears that the Tomato here also is occurring in blood vessels, so the authors should include an IHC of an endothelial cell marker (as above).

5. Results: It is perplexing that most of the RosaGreen GFP-labeled cells are Iba1-NeuN-GFAP-. The stitched image in Fig. 1-1A makes it seem as though there may be large deposits of GFP in the brain that are larger than any single cell. This appears as though it could be the result of secondary antibody deposition if these brains were stained for amplification of the GFP signal. Did you also confirm that this was true for the endogenous GFP signal in an unstained brain? The authors should consider the possibility of secondary antibody deposition or fluorophore conglomerates in their results and/or discussion.

6, Results Moreover, the Cx3cr1-GFP mouse line did now show any leakage of the GFP reporter into neurons and astrocytes.... now should be NOT.

7. I suggest to substitute general “excitatory injury” to more specific “status epilepticus”, if not all, at least, in the Result section.

8. Please provide actual % (i.e., quantitative data) in the text of Results, rather than ‘most cells’
