# Supplementary figures and images for: Targeting Microglia Using Cx3cr1-Cre Lines: Revisiting the Specificity
Source: eNeuro. 2019 Jul 8;6(4):ENEURO.0114-19.2019. doi: 10.1523/ENEURO.0114-19.2019 (PMC6620394; doi:10.1523/ENEURO.0114-19.2019)

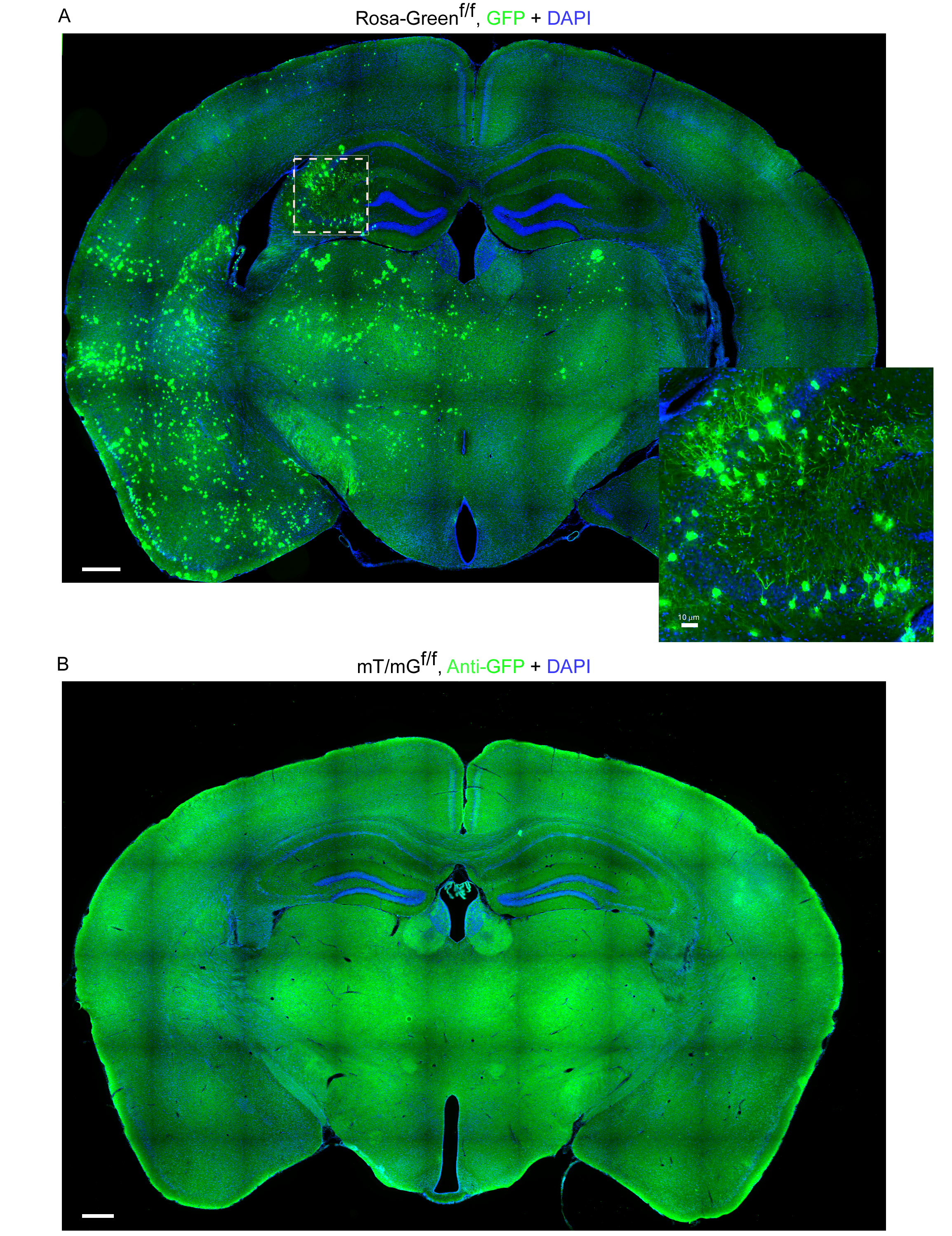

Supplement: Extended Data Figure 1-1 — Evaluation of the cre-independent fluorescent protein expression of reporter lines. A, B, Full montage images of brain coronal sections showing GFP expression in the Rosa-Greenf/f and mT/mGf/f mouse lines. The Rosa-Greenf/f mouse brain sections were stained with DAPI. The mT/mGf/f mouse brain sections were stained with rabbit anti-GFP (green) and DAPI. Scale bar, 200 μm. Download Figure 1-1, TIF file. [file sup_enu-eN-MNT-0114-19-s04.tif]

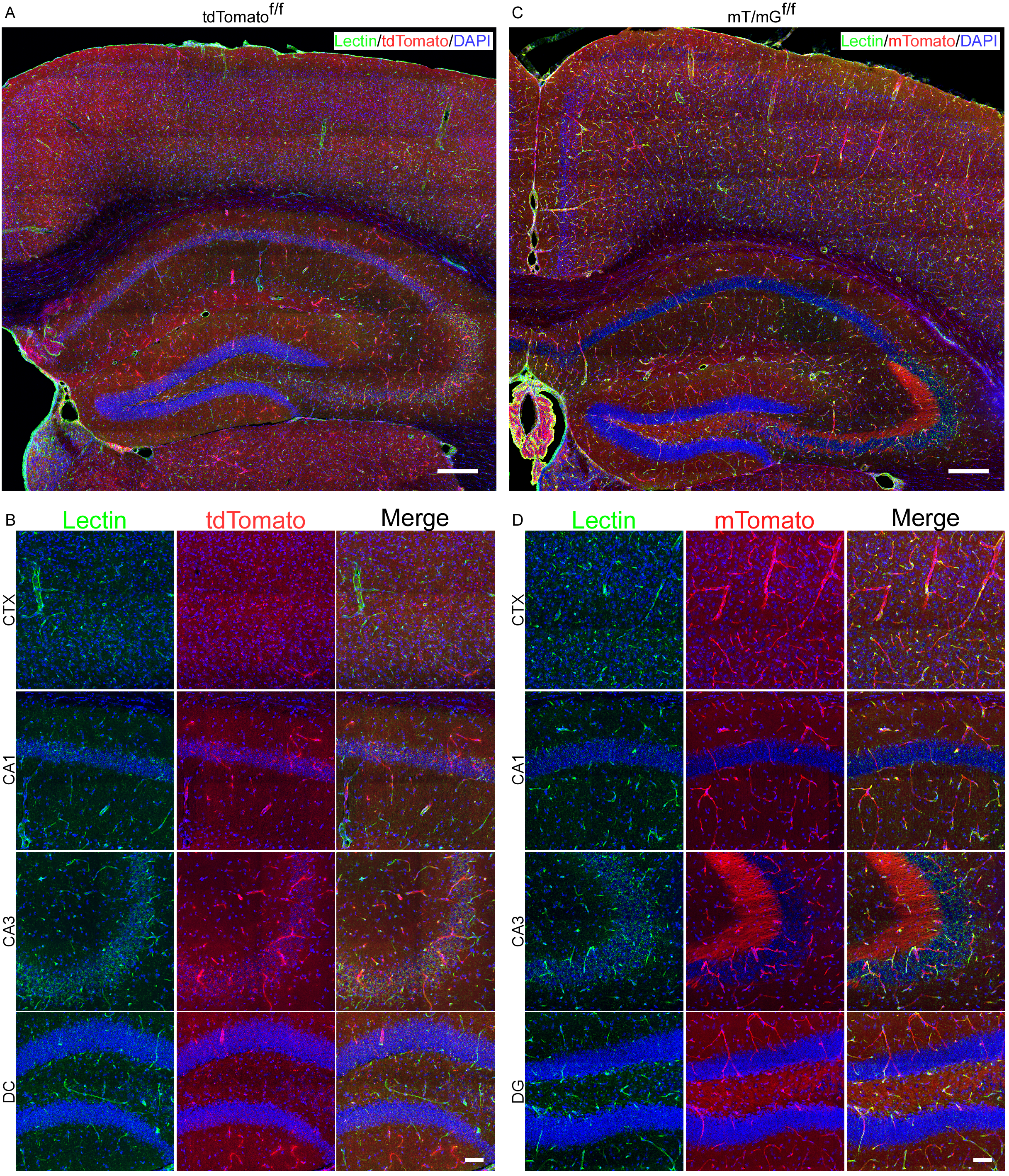

Supplement: Extended Data Figure 1-2 — Fluorescent protein expression in blood vessels in mT/mGf/f and tdTomatof/f lines. A, Full montage of confocal images showing mTomato expression in the CTX and the hippocampus of mT/mGf/f mice, respectively. Brain sections were stained with lectin (green) and DAPI (blue). Scale bar, 200 μm. B, High-magnification images from CTX, CA1, CA3, and DG. Scale bar, 20 μm. C, Full montage of confocal images showing tdTomato expression in the CTX and the hippocampus of tdTomatof/f mice, respectively. Brain sections were stained with lectin (green) and DAPI (blue). Scale bar, 200 μm. D, High-magnification images from CTX, CA1, CA3, and DG. Scale bar, 20 μm. Download Figure 1-2, TIF file. [file sup_enu-eN-MNT-0114-19-s05.tif]

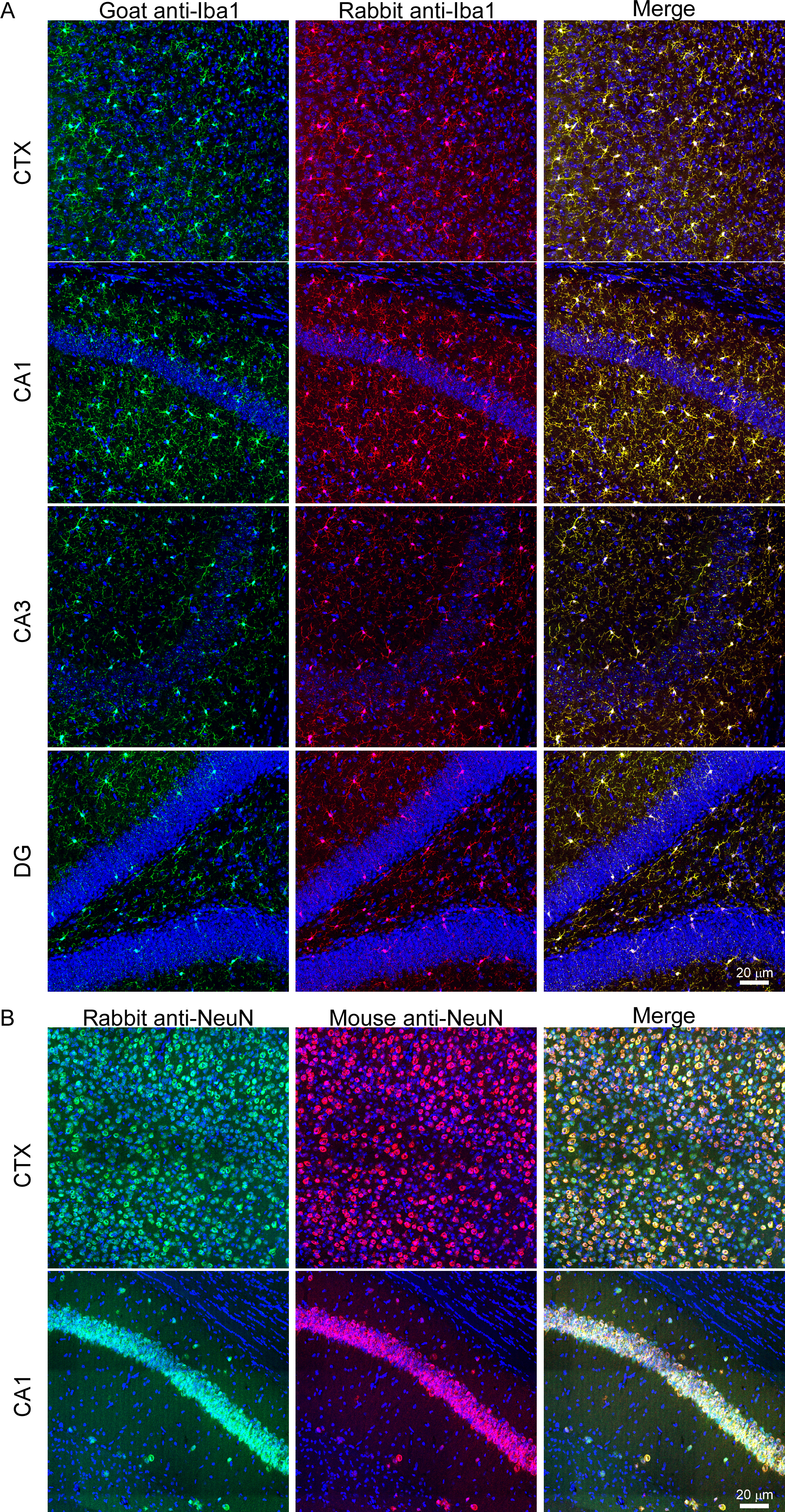

Supplement: Extended Data Figure 1-3 — Specificity of anti-Iba1 and NeuN antibodies used in this study. A, Confocal images showing both goat anti-Iba1 (green) and rabbit anti-Iba1 (red) antibodies can selectively label microglia in control mice. B, Confocal images showing both mouse anti-NeuN (red) and rabbit anti-NeuN (green) antibodies can selectively label neurons in control mice. Download Figure 1-3, TIF file. [file sup_enu-eN-MNT-0114-19-s01.tif]

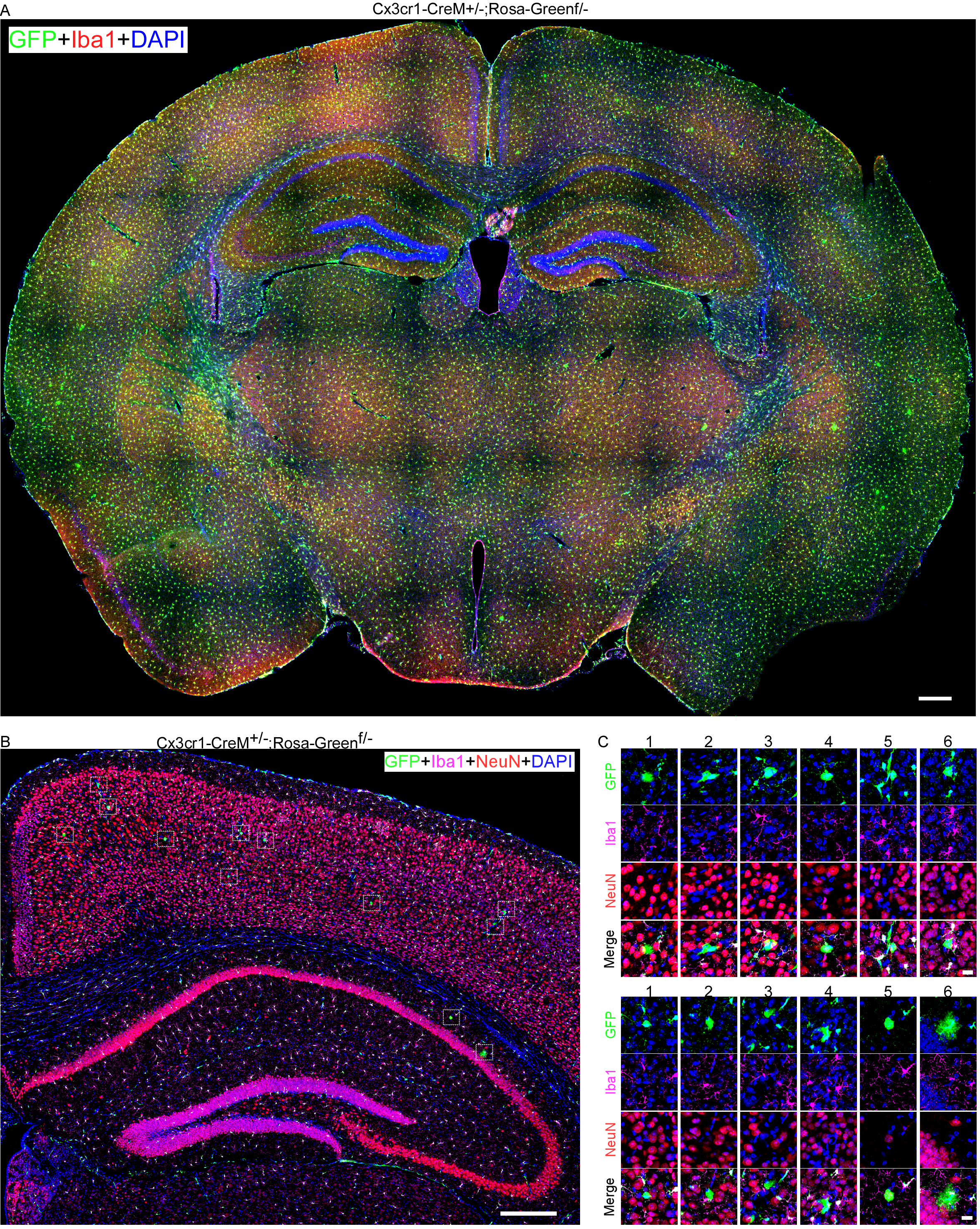

Supplement: Extended Data Figure 2-1 — Specificity of the Cx3cr1-creM+/- mouse line. A, Full montage of brain coronal sections showing GFP reporter expression in microglia in Cx3cr1-creM+/-;Rosa-Greenf/- mice. Mouse brain sections were stained with goat anti-Iba1 (red) and DAPI (blue). Scale bar, 200 μm. B, Brain sections of Cx3cr1-CreM+/-;Rosa-Greenf/- mice were stained with: goat anti-Iba1 (pink), rabbit anti-NeuN (red), and DAPI (blue). Confocal images showing bright, large GFP-positive cells (numbered) that were sporadically present in the cortex and hippocampus. Scale bar, 200 μm. C, High-magnification images of GFP+ cells showing that only one out of twelve GFP+ cells co-localized with NeuN+ (red) cells. Scale bar, 20 μm. Download Figure 2-1, TIF file. [file sup_enu-eN-MNT-0114-19-s02.tif]
